# The epidemiology and clinical features of HIV and *Trypanosoma cruzi* (Chagas disease) co-infection: A systematic review and individual patient data analysis

**DOI:** 10.1371/journal.pntd.0012808

**Published:** 2026-01-22

**Authors:** Natalie Elkheir, Jessica Carter, Catherine Dominic, Pat Lok, Temitope Fisayo, Melina Michelen, Barbara De Barros, Jaimie Wilson Goldsmith, Michael Butler, Amy Price, Anushka Mehotra, Laura Nabarro, Nadia Ahmed, Peter L. Chiodini, David A. J. Moore

**Affiliations:** 1 Clinical Research Department, London School of Hygiene & Tropical Medicine, London, United Kingdom,; 2 Hospital for Tropical Diseases, University College London Hospitals, London, United Kingdom; 3 UK Chagas Hub, United Kingdom; 4 City St George’s, University of London, London, United Kingdom; 5 Queen Mary’s University of London, London, United Kingdom; 6 Norfolk and Norwich University Hospitals NHS Trust, Norwich, United Kingdom; 7 Anglia Ruskin University, Chelmsford, United Kingdom; 8 University College London Hospitals NHS Trust, London, United Kingdom; 9 School of Health Sciences, City University of London, London, United Kingdom; 10 Royal Free London NHS Foundation Trust, London, United Kingdom; 11 Imperial College Healthcare Trust, London, United Kingdom,; 12 North West University Healthcare NHS Trust, London, United Kingdom; 13 One Health Lewisham (GP Federation), London, United Kingdom; 14 Central and North West London NHS Foundation Trust, London, United Kingdom; Centro de Pesquisa Gonçalo Moniz-FIOCRUZ/BA, BRAZIL

## Abstract

**Background:**

Narrative descriptions of HIV and *Trypanosoma cruzi,* the causative agent of Chagas disease, co-infection exist in the literature but the breadth and depth of the data underlying these descriptions has not been previously thoroughly scrutinised and reactivation is poorly understood. The aim of this systematic review was to identify, synthesise and analyse the published literature on the epidemiology and clinical features of *T. cruzi* and HIV co-infection.

**Methods:**

A systematic review of published literature on HIV and *T. cruzi* co-infection was conducted. Six international databases were searched: Medline, Embase, Global Health, Global Index Medicus (including LILACS, AIM, IMEMR, IMSEAR & WPRIM), Web of Science and Scopus. Articles reporting on HIV and *T. cruzi* co-infection, as defined by the authors, with no restrictions on study type, language or date of publication or reporting were included.

**Results:**

152 articles (62% case reports or series) were included, of which 110 reported individual patient data on 352 individuals with HIV and *T. cruzi* co-infection. Reported prevalence of co-infection varied by region and setting of screening, ranging from 0.2% to 5%. 86% of reactivations were reported in individuals with CD4 < 200 cells/mm^3^. CNS reactivation, typically presenting with meningoencephalitis and/or central nervous system (CNS) lesions, accounted for 85% of all published cases of reactivation. Myocarditis (accounting for 10% published reactivation cases) was less well characterised. Mortality of all reactivation cases was 67% (79% in those with CNS reactivation).

**Conclusion:**

*T. cruzi* reactivation mainly affects those with untreated HIV and lower CD4 counts. CNS reactivation is the most common clinical picture and confers high mortality. Prompt recognition of reactivation and immediate initiation of trypanocidal therapy (with benznidazole or nifurtimox) is recommended. Increased education and better awareness of the risks of co-infection are needed, as is systematic screening of individuals at-risk.

**Trial registration:**

Prospero CRD42020216125

## Author summary

The consequences of infection with both HIV and *Trypanosoma* cruzi (the parasite which causes Chagas disease) have been written about in the literature since the 1990s. In this review article, the evidence on co-infection is systematically identified, summarized and critiqued. Most evidence comes from small case reports or series of individual patients and is not of high quality. The main risk of co-infection is reactivation in the central nervous system, when the *T. cruzi* parasite replicates, causing meningoencephalitis. This usually occurs when HIV is not well-controlled and is associated with a high risk of death. Prompt recognition of co-infection, and antiparasitic treatment of *T. cruzi* can mitigate this risk.

## Introduction

Chagas disease is one of the most important zoonotic infectious diseases in Latin America and is increasingly being recognised as a public health problem in non-endemic settings within migrants from Latin America [1–3]. It is caused by the protozoan parasite *Trypanosoma cruzi* and is endemic in the 21 countries of Central America, South America and Mexico. About 7 million people worldwide are estimated to be infected with *T. cruzi* and approximately 12,000 people die each year from clinical manifestations of Chagas disease [4]. Infection is believed to be lifelong without treatment. The consequences of this chronic infection include progression to determinate cardiac or gastrointestinal end-organ disease, which affects approximately one third of individuals with *T. cruzi*. Chronic infection also poses an additional risk of parasite reactivation and replication in the context of immunocompromise such as immunosuppressant medication or HIV.

The first recognised description of *T. cruzi* reactivation in people living with HIV (PLHIV) was reported in Argentina in 1990, in a patient with central nervous system reactivation [5]. Since then, many articles have reported high mortality rates associated with HIV/*T. cruzi* co-infection [6–8]. A systematic review including articles published until 2010 identified 291 cases of co-infection published in the literature, and 100% mortality in untreated cases of reactivation [9]. A narrative review published in 2021 described the epidemiology of co-infection, the clinical presentations most commonly associated with *T. cruzi* reactivation in PLHIV and highlighted the many gaps in knowledge [10]. This systematic review will complement these earlier papers, through drawing together all international literature and publicly available data to provide an analysis of the current state of knowledge.

### Aims and objectives

The aim was to systematically identify, synthesise and evaluate the evidence on the epidemiology and clinical features of HIV and *T. cruzi* co-infection, with a view to informing clinical and public health management, screening policy, and identify research priorities.

## Methods

A systematic review was conducted according to PRISMA guidelines [11], and registered with the International Prospective Register of Systematic Reviews (PROSPERO ID: CRD42020216125).

### Eligibility criteria

***Inclusion criteria:*** This review includes articles reporting on HIV and *T. cruzi* co-infection, as defined by the authors, with no restrictions on study type, language or date of publication or reporting.
***Exclusion criteria:*** Editorials and opinion papers were excluded, along with any articles not presenting primary data. Studies that included people with HIV and *T. cruzi* as a sub-group but did not present disaggregated data for any of the outcome measures of interest were excluded, unless corresponding authors were able to provide the required information (following two email attempts to contact authors). Articles where HIV and *T. cruzi* prevalence were reported separately (mainly with reference to blood bank screening and with no way to infer co-infection) were excluded. Systematic reviews and reviews were excluded, but citation tracking of any such publications was undertaken to identify additional publications not otherwise identified by the search strategy.

### PICO question

Population: People of any age with *T. cruzi* infection/Chagas disease
Exposure: HIV co-infection, as defined by authors
Comparison: N/A
Outcomes:
  1. Prevalence of co-infection
    - Characteristics of patients and co-infection, including but not limited to:
    - Age
    - Sex
    - Country of birth/residence
    - CD4 count and HIV viral load
    - Clinical presentation
    - Presence of other opportunistic infections
    - Setting of diagnosis and method of diagnosis (e.g., clinical, serological, parasitological)
    - Clinical investigations (including bloodwork, imaging and cerebrospinal fluid examination)
    - Treatment (antiretroviral, antiparasitic therapy and other)
    - Clinical outcomes: measures of morbidity and mortality

### Information sources

The following six databases were searched on 1^st^ July 2022: Ovid Medline, Ovid Embase, Ovid Global Health, Global Index Medicus (including LILACS, AIM, IMEMR, IMSEAR & WPRIM), Web of Science and Scopus. For any review articles identified, the listed references were screened, and citations of the articles were reviewed to identify any additional papers not captured by the original search strategy. Authors were contacted to request additional data where data was missing.

### Search strategy

Controlled subject headings and keywords of the following concepts were searched:

1) HIV

And

2) Chagas disease or *Trypanosoma cruzi* infection

There were no limits on language or date of publication.

### Selection process

Title and abstract screening of all records identified by the search strategy was carried out independently by two reviewers (NE and JC). Subsequent full-text screening of selected articles against the inclusion criteria was carried out independently by the same two reviewers, with all discrepant assessments resolved by consensus discussion. The Rayyan systematic review data automation tool was used to organize articles and record screening decisions.

### Data collection process

Data were managed using Endnote for de-duplication of articles, Rayyan for screening, Microsoft Excel for data extraction and STATA version 18 for data analysis. The data extraction tool was developed in Microsoft Excel and piloted with input from the study team. Following this pilot assessment, the data extraction tool was split into two: one for articles that presented only aggregate-level data (e.g., prevalence studies) and one for articles with individual-level data (predominantly case reports and case series). Data extraction was performed independently by two reviewers (NE and either CD, PL, TF, MM, BdB, JWG, MB, AP or AM), who were fluent or native in the language of the article. Data extraction was checked by a third reviewer (JC), using the DeepL Pro (Cologne, Germany) online translation tool if necessary. Disagreements on interpretation of data to extract were resolved, and consensus reached, through discussion. Where important data was missing, the corresponding authors of the articles were contacted (twice via email) to request it.

### Data items

The following data (where available) was extracted from all articles: study design, aim, country of study, setting and context, inclusion and exclusion criteria, study population size (including prevalence of co-infection where reported) and characteristics, diagnostic criteria for HIV and *T. cruzi* infection, CD4 count, HIV viral load, clinical syndromes (signs and symptoms), other opportunistic infections reported, investigations (including laboratory and radiological findings), treatments (antiretroviral, antiparasitic and others) and clinical outcomes (measures of morbidity and mortality).

### Study risk of bias assessment

The Joanne Briggs Institute Critical Appraisal Checklists [12] were used to assess for risk of bias in the studies included in the review. Two reviewers (NE and JC) assessed for risk of bias independently and reached consensus through discussion where disagreements arose. No formal measures to assess reporting bias or certainty in the body of evidence were taken, although these are qualitatively described.

### Effect measures

Prevalence of co-infection in the study population was extracted where available (for both HIV amongst subjects with *T. cruzi* infection and for *T. cruzi* infection amongst subjects with HIV), and the range of estimates were presented according to study setting. For studies reporting individual-level patient data, mortality was calculated as an overall proportion.

### Synthesis methods

Articles were analysed in three groups: those that presented prevalence data, those that presented aggregate-level data of selected populations, and those that presented any individual patient-level data. The inclusion criteria for prevalence studies (based on Joanna Briggs Institute guidance [13]) were as follows: cross-sectional study design, population samples of more than 50 individuals (of any age, sex, geographical location or sociodemographic characteristics), studies in unselected populations (or populations recruited based on either known HIV or *T. cruzi* infection), and prevalence of co-infection reported. The exclusion criteria for prevalence studies were: hospitalised patients, populations selected based on characteristics other than HIV or *T. cruzi* status, such as study samples drawn from populations with known comorbidities or undergoing interventions that put them at increased risk of co-infection. Articles that met these prevalence study criteria were summarised in Table 1 according to the setting (and context) of presentation. A meta-analysis of prevalence estimates was not performed due to the substantial heterogeneity among the different contexts.

**Table 1:** Study characteristics and profile of HIV/*T. cruzi* co-infection in articles presenting prevalence of co-infection (in unselected, non-hospitalised patients).

Author, year	Country	Study design	Population description	Co-infection/total P^*^	Prevalence
***Screening people not known to have HIV or T. cruzi infection***					
Aguiar, 1999 [14]	Brazil	Cross-sectional	First time blood donors to blood bank in Mato Gross do Sul State all tested for HIV, *T. cruzi*, hepatitis B and C, and syphilis.	1/476	0.002
***Testing for T. cruzi infection in people with known HIV***					
***Chagas-endemic countries***					
Almeida, 2010 [15]	Brazil	Cross-sectional	*T. cruzi* seroprevalence study of PLHIV under (outpatient) care *(IPD n = 20, 9 from cross-sectional study plus 11 from clinics)*	9/716	0.013
Stauffert, 2017 [16]	Brazil	Cross-sectional	Seroprevalence of *T. cruzi* in PLHIV under (outpatient) care in one clinic in Rio Grande do Sul State	10/200	0.05
***Non-endemic countries***					
Llenas Garcia, 2012 [26]	Spain	Cross-sectional	*T. cruzi* screening in Latin American migrants under (outpatient) care in HIV clinic *(IPD n = 3)*	4/154	0.026

* = Number with co-infection / total (at-risk) study population. **Articles that reported any Individual patient data (IPD) are indicated, along with number of patients (n) with extractable data included in individual patient analysis and therefore Table 2.

† = People living with HIV.

Articles that presented aggregate data on co-infection in selected populations that did not meet the above prevalence study criteria were summarised thematically, according to study design and setting, in Table 2.

**Table 2:** Study characteristics and profile of HIV/*T. cruzi* co-infection in articles of selected populations presenting aggregate data.

Author, year	Country	Study design	Population description	n co-infected/study population
***Screening people not known to have HIV or T. cruzi infection***				
Carneiro-Proietti, 1998 [27]	Brazil	Cross-sectional	Patients with haemophilia	2/226
Auger, 2002 [17]	Argentina	Cross-sectional	Patients seen in emergency department in Buenos Aires (IPD** n = 2)	2/1680
Bocanegra, 2014 [18]	Spain	Cross sectional	Migrants from Chagas-endemic countries attending tropical medicine unit in Barcelona	4/416
***Testing for T. cruzi infection in people with known HIV***				
***Chagas-endemic countries***				
Spina-França, 1988 [28]	Brazil	Cross-sectional	PLHIV^†^ with neurological presentations and CSF^‡^ to hospital *(IPD n = 1)*	1/200
Livramento, 1989 [29]	Brazil	Cross-sectional	PLHIV with neurological presentations and CSF^‡^ analysis *(IPD n = 2)*	2/170
Torrealba, 1990 [30]	Chile	Case-series	PLHIV with neurological presentation and brain biopsy *(IPD n = 1)*	1/4
Reiche, 1996 [31]	Brazil	Cross-sectional	HIV reactive samples (selected by random from the lab, from patients who had attended hospital in Londrina, Brazil) tested for *T. cruzi*	4/181
Lazo, 1998 [32]	Brazil	Case-series	PLHIV with encephalitis *(IPD n = 15)*	15/22
Oelemann, 2000 [33]	Brazil	Cross-sectional	PLHIV with chronic diarrhoea *(IPD n = 4)*	4/95
Scapellato, 2001 [34]	Argentina	Cross-sectional	Retrospective review of records of PLHIV attending Buenos Aires Hospital and routine tests performed for co-infection, including *T. cruzi*	8/184
Rodrigues, 2005 [35]	Brazil	Cross-sectional	PLHIV with and without *T. cruzi* infection, assessing cytokine serum levels	18/28
Scapellato, 2006 [36]	Argentina	Cross-sectional	*T. cruzi* seroprevalence in PLHIV who inject drugs	3/328
Warley, 2010 [37]	Argentina	Cross-sectional	PLHIV on ART^§^ with malignancy or infection	4/235
Reimer-McAtee, 2014 [38]	Bolivia	Cross-sectional	PLHIV presenting to hospital and use of PCR	38/133
Bowman, 2015 [39]	Bolivia	Cross-sectional	PLHIV hospitalised in Santa Cruz	37/157
Benchetrit, 2017 [40]	Argentina	Cross-sectional	*T. cruzi* seroprevalence in PLHIV + MSM^‖^, STI^¶^ clinic attendees & IVDU^#^	8/280
Ignacio-Junior, 2018 [41]	Brazil	Cross-sectional	*T. cruzi* seroprevalence of PLHIV seen at an infectious diseases unit in Sao Paulo	2/240
Dias, 2018 [19]	Brazil	Cross-sectional	Review of medical records of PLHIV seen in outpatient clinic in Goias state	1/539
Reimer-McAtee, 2021 [20]	Bolivia	Cross-sectional	*T. cruzi* PCR in PLHIV in hospital in Cochabamba *(IPD n = 4, all with reactivation)*	32/116
***Non-endemic countries***				
Rodríguez-Guardado, 2009 [25] ^††^	Spain	Cross-sectional	*T. cruzi* screening in Latin American migrant PLHIV *(IPD n = 2)*	2/19
Rodríguez-Guardado, 2010 [42] ^††^	Spain	Cross-sectional	*T. cruzi* screening in Latin American migrant PLHIV	2/19
Rodríguez-Guardado, 2012 [43] ^††^	Spain	Cross-sectional	*T. cruzi* screening in Latin American migrant PLHIV	2/19
Salvador, 2013 [44]	Spain	Cross-sectional	*T. cruzi* screening in migrant PLHIV who attended Infectious Diseases Department in Barcelona	5/126
Ahmed, 2016 [45]	UK	Cross-sectional	*T. cruzi* screening in Latin American PLHIV in sexual health clinic *(IPD n = 1)*	1/40
Evans, 2018 [24]	US	Cross-sectional	Retrospective data analysis *of T. cruzi* screening results in Latin American migrant PLHIV *(IPD n = 2)*	2/179
Rodari, 2022 [46]	Italy	Cross-sectional	*T. cruzi* seroprevalence in Latin American migrant PLHIV enrolled in a cohort study of ART§ naïve patients (stored sera samples tested).	5/389
***Testing for HIV in people with known T. cruzi infection***				
***Chagas-endemic countries***				
Bisugo, 1998 [47]	Brazil	Cross-sectional	Patients with known *T. cruzi* seen in hospital	32/78
Perez-Ramirez, 1999 [22]	Brazil	Cross-sectional	Patients with known *T. cruzi* in four hospitals *(IPD n = 33)*	34/71
Sartori, 2002 [21]	Brazil	Cross-sectional	Patients with known *T. cruzi* attending infectious diseases and HIV clinics	29/110
Birrer, 2021 [48]	Brazil	Cross-sectional	Blood donors to clinic in Brazil that were selected based on inconclusive or reactive *T. cruzi* serology at triage	1/295
***Non-endemic countries***				
Ramos, 2009 [49]	Spain	Cross-sectional	Series of patients with known *T. cruzi* seen in three hospitals in Alicante	1/67
Escoin, 2011 [50]	Spain	Cross-sectional	Series of patients with known *T. cruzi* seen at tertiary centre screened for HIV	2/282
Salvador, 2015 [51]	Spain	Cross-sectional	Retrospective review of medical records of patients with *T. cruzi* in three hospitals in Catalonia *(IPD n = 12)*	12/1823
Ramos-Rincon, 2021 [52]	Spain	Cross-sectional	All patients discharged from a hospital with *T. cruzi*	45/5022
***Death certificates, registries & reports on co-infection***				
Rocha, 1994 [53]	Brazil	Cross-sectional	Autopsies of patients with HIV/*T. cruzi* co-infection	23/23
Martins Melo, 2012 [8]	Brazil	Cross-sectional	Death certificates, all deaths in Brazil 1999–2007	74/8942217
Garcia-Pino, 2014 [54]	Colombia	Cross-sectional	Autopsies and DNA analysis of PLHIV	6/155
Melo-Uribe, 2014 [55]	Colombia	Cross-sectional	Autopsies of PLHIV and opportunistic infections	<20/155
Gattoni, 2015 [56]	Brazil	Cross-sectional	Autopsies of PLHIV with opportunistic infections (including Chagas reactivation)	2/326
Hernandez, 2017 [57]	Colombia	Cross-sectional	Autopsies of PLHIV and opportunistic infections in Santander	13/3497
Olivera, 2021 [58]	Colombia	Cross-sectional	Death reports in Colombia of patients with known *T. cruzi* 1979–2018	95/3276
Martins Melo, 2022 [23]	Brazil	Cross-sectional	Death certificates all deaths in Brazil, 2000–2019	196/22663092
***Studies of known co-infection***				
Braz, 2001 [59]	Brazil	Cross-sectional	*T. cruzi* parasitaemia in immunocompromised patients	–
Sartori, 2007 [60]	Brazil	Cohort	Observational cohort of known co-infection 1989–2005	53/53
Lages-Silva, 2009 [61]	Brazil	Cross-sectional	*T. cruzi* genetic characterization in +/- HIV, + /- co-infection & + /- reactivation	38/91
Scapellato, 2009 [62]	Argentina	Cohort	Mother to child transmission of *T. cruzi* (3/94 mothers co-infected with HIV)	3/94
De-Freitas, 2011 [63]	Brazil	Cross-sectional	PCR, blood culture and xenodiagnosis in patients with known co-infection	32/91
Shikanai-Yasuda, 2014 [64]	Brazil	Cohort	Parasitaemia in immunosuppressed patients, including co-infection	43/43
Castro-Sesquen, 2016 [65]	Bolivia	Case-control	Nanotechnology in urine in patients with HIV/*T. cruzi* co-infection	20/39
Fernandez, 2015 [66]	Argentina	Cross-sectional	Known HIV/*T. cruzi* co-infection with reactivation at Buenos Aires Hospital	23/23
Tozetto-Mendoza, 2017 [67]	Brazil	Cross-sectional	Cytokine producing T cells in Chagas, co-infection, HIV and healthy controls	11/35
Fernandez, 2018 [68]	Argentina	Case-series	Known HIV *T. cruzi* co-infection with neuro-reactivation in hospital	7/7
Shikanai-Yasuda, 2021 [69]	Multiple	Cross-sectional	Clinical profile and mortality of co-infection from multi-centre network	241/241

* Articles that reported any Individual patient data (IPD) are indicated, along with number of patients (n) with extractable data included in individual patient analysis and therefore Table 2.

† = People living with HIV, ‡ = Cerebrospinal fluid, § = Antiretroviral therapy, ‖ = Men who have sex with men, ¶  = Sexually-transmitted infection, # = Intravenous drug user, †† = duplicated data therefore the article with more complete dataset included in IPD analysis, - = not applicable

Articles with individual-level data (summarised in Table 3) were assessed for duplication (i.e., the same cases being published more than once) by checking the epidemiological and clinical details of age- and sex- matched cases. Duplicate cases are still presented in Table 3 but are excluded from the analysis (and the estimate of number of total cases published in the literature). Once duplicate cases were excluded, due to heterogeneity in reporting of reactivation, the study team classified clinical presentations reported in articles into phenotypic groups (according to *T. cruzi* laboratory findings and clinical presentation, described further below and in Fig 3) by consensus. The individual patient analysis focussed on individuals meeting the criteria for reactivation.

**Table 3:** Study characteristics of articles presenting individual patient data on HIV/*T. cruzi* co-infection.

Author, year	Country	n	Author, year	Country	n	Author, year	Country	n
***Endemic countries***			Oelemann, 2000 [33]	Brazil	4	Castro-Sesquen, 2016 [70]	Bolivia	31
Spina-França, 1988 [28]	Argentina	1	Lages-Silva, 2002 [71]	Brazil	1	Simioli, 2016 [72]	Argentina	1
Livramento, 1989 [29]	Brazil	2	Chirino, 2001 [73]	Venezuela	1	Fica, 2017 [74]	Chile	1
Del Castillo, 1990 [5]	Argentina	1	Auger, 2002 [17]	Argentina	2	Lopez, 2018 [75]	Argentina	1
Torrealba, 1990 [30]	Brazil	1	Santos, 2002 [76]	Brazil	1	Benchetrit, 2018 [77]	Argentina	9
Ferreira, 1991 [78]	Brazil	1	Rodrigues, 2002 [79]	Brazil	1	Chalub, 2019 [80]	Argentina	1
Gallo, 1992 [81]	Brazil	1	Sartori, 2002 [82]	Brazil	1	Guidetto, 2019 [83]	Argentina	2
Oddo, 1992 [84]*	Chile	2	Antunes, 2002 [85]	Brazil	1	Reimer-McAtee, 2021 [20]	Bolivia	4
Cardozo, 1992 [86]	Uruguay	1	Marques De Brito, 2003 [87]	Brazil	2	Fernández, 2021 [88]	Argentina	6
Rosemberg, 1992 [89]	Brazil	1	Rotta, 2003 [90]	Brazil	1	Muñoz-Calderón, 2022 [91]	Argentina	3
Rocha, 1993 [92]	Brazil	1	Madalosso, 2004 [93]	Brazil	1	Marcon, 2022 [94]	Brazil	8
Libaak, 1993 [95]	Argentina	1	Da-Cruz, 2004 [96]	Brazil	3	Bowman, 2022 [97]	Bolivia	4
Metze, 1993 [98]	Brazil	1	Auger, 2005 [99]	Argentina	8	Total no. patients in endemic settings*		315
Nishioka, 1993 [100]	Brazil	1	Burgos, 2005 [101]	Argentina	1	***Non-endemic countries***		
Solari, 1993 [102]	Chile	1	Valergaa, 2005 [103]	Argentina	1	Gluckstein, 1992 [104]	US	1
Freilij, 1995 [105]	Argentina	2	Corti, 2006 [106]	Argentina	1	Rivera J, 2004 [107]	US	1
Sartori, 1995 [108]*	Brazil	1	Salcedo, 2006 [109]	Mexico	1	Yoo, 2004 [110]	US	1
Freilij, 1995 [111]	Argentina	3	Sartori, 2007 [60]*	Brazil	53	Lury, 2005 [112]	US	1
Pimentel, 1996 [113]	Brazil	1	Burgos, 2008 [114]	Argentina	1	Lambert, 2006 [115]	US	1
Di Lorenzo, 1996 [116]	Argentina	2	Sica, 2008 [117]	Argentina	3	Rodríguez-Guardado, 2009 [25]	Spain	2
Ferreira, 1997 [118]	Brazil	3	Cordova, 2008 [119]	Argentina	15	Verdú, 2009 [120]	Spain	1
Montero, 1998 [121]	Argentina	2	Diazgranados, 2009 [122]	Colombia	1	Campo, 2010 [123]	US	1
Iliovich, 1998 [124]	Argentina	1	De Almeida, 2009 [125]	Brazil	1	Rodríguez-Guardado, 2011 [126]	Spain	1
Pacheco, 1998 [127]	Brazil	1	Moretta, 2009 [128]	Argentina	1	Llenas-Garcia, 2012 [26]	Spain	3
Cohen, 1998 [129]	Argentina	1	De Almeida, 2009 [130]	Brazil	2	Angheben, 2013 [131]	Italy	1
Sartori, 1998 [132]*	Brazil	18	Rodríguez, 2009 [133]	Colombia	1	Mina, 2013 [134]	Italy	1
Sartori, 1998 [135]*	Brazil	1	Rocha, 2010 [136]	Brazil	1	Yasukawa, 2014 [137]	US	1
Lazo, 1998 [32]	Brazil	15	Lopez, 2010 [138]	Chile	1	Salvador, 2015 [51]	Spain	12
Galhardo, 1999 [139]	Brazil	1	Menghi, 2011 [140]	Argentina	1	Martinez-Ales, 2016 [141]	Spain	1
dos Santos, 1999 [142]	Brazil	1	Catay, 2010 [143]	Argentina	1	Alyemni, 2016 [144]	US	1
Filho, 1999 [145]	Brazil	1	Almeida, 2010 [15]	Brazil	20	Ahmed, 2016 [45]	UK	1
Pagano, 1999 [146]	Argentina	10	Agosti, 2012 [147]	Argentina	2	Shah, 2017 [148]	US	1
Silva, 1999 [149]	Brazil	5	Bisio, 2013 [150]	Argentina	1	Gomez, 2018 [151]*	US	1
Sartori, 1999 [152]*	Brazil	1	Castrillon, 2013 [153]	Argentina	1	Evans, 2018 [24]	US	2
Nisida, 1999 [154]	Brazil	4	Hernández, 2014 [155]	Colombia	1	Multani, 2019 [156]*	US	1
Perez-Ramirez, 1999 [22]	Brazil	33	Spadafora, 2014 [157]	Venezuela	1	Bosch-Nicolau, 2019 [158]	Spain	1
Concetti, 2000 [159]	Argentina	1	Uliarte, 2015 [160]	Argentina	1	Bustamante, 2020 [161]	US	1
Corti, 2000 [162]	Argentina	1	Bucchieri, 2015 [163]	Brazil	1	Total no. patients non-endemic settings*		37

* These articles reported duplicated cases. The article with more complete data was retained in the analysis of individual patient data (and in the estimate of total number of patients), and the less complete article excluded.

Continuous data for all patients were presented as the mean and standard deviation (if normally distributed) or median and interquartile range (if not), and comparisons between different clinical presentations of reactivation were assessed using the Student’s t-test or Wilcoxon rank-sum test respectively. The categorical data for all patients are presented as counts and frequencies, and comparisons were performed using the Chi-squared or Fisher’s exact test, as appropriate. All analyses were conducted using STATA version 18 and a *P* value of less than 0.05 was considered statistically significant.

## Results

### Study selection

**Fig 1:**
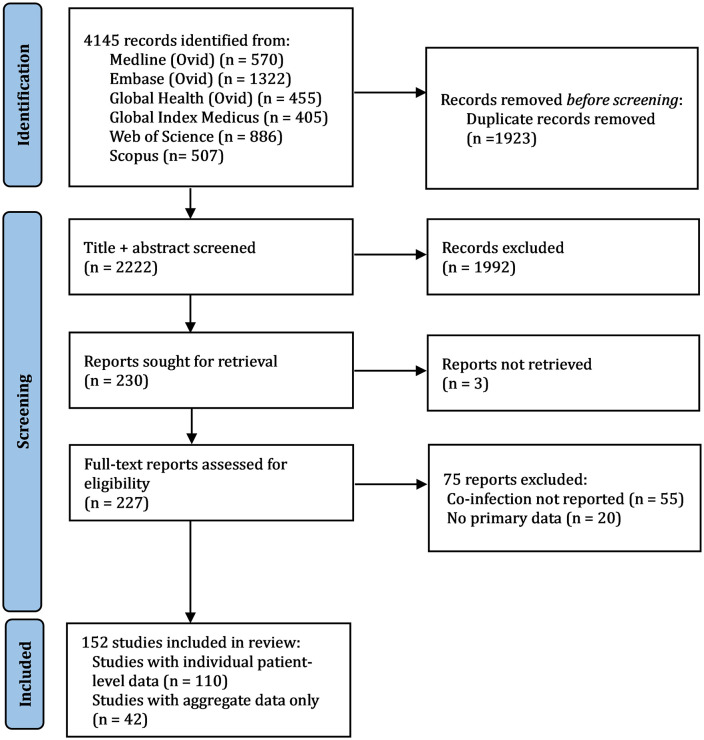
PRISMA (Preferred Reporting Items for Systematic reviews and Meta-Analyses) flow diagram outlining study selection. Database searches were performed up to 1^st^ July 2022.

Ovid Medline, Ovid Embase, Ovid Global Health, Global Index Medicus (including LILACS, AIM, IMEMR, IMSEAR & WPRIM), Web of Science and Scopus were searched (as per methods section) on 1^st^ July 2022. Fig 1 outlines the process from article identification to inclusion.

### Study characteristics

152 articles were included in this systematic review, of which 131 (86%) were journal articles, 20 (13%) were conference abstracts or posters and one (1%) an online report. These articles consist of case reports (n = 68, 45%), case series (n = 26, 17%), cross-sectional studies (n = 51, 34%), cohort studies (n = 5, 3%) and case-control studies (n = 2, 1%).

Of these 152 studies included, 110 reported individual patient data on 352 individuals with HIV and *T. cruzi* co-infection (once duplicate cases were identified based on demographic and clinical features, and were excluded from the analysis).

**Fig 2:**
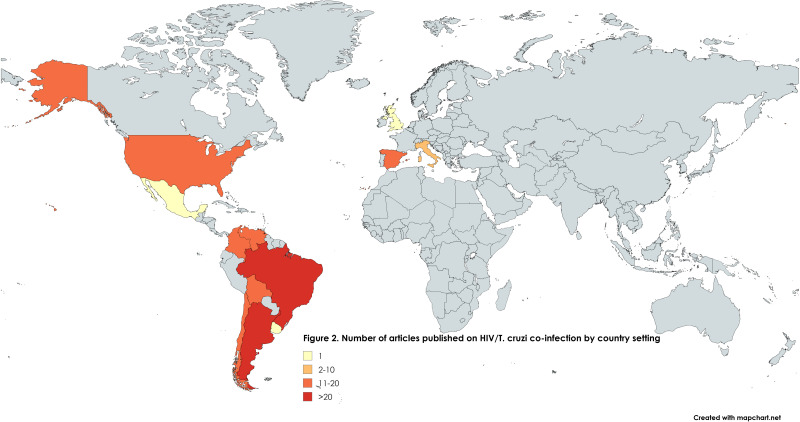
Map generated from mapchart.net and licensed under a Creative Commons Attribution-ShareAlike 4.0 International License.

121 studies were performed in endemic countries (Fig 2): Brazil (n = 60, 39% of all included articles), Argentina (n = 39, 26%), Colombia (n = 7) Bolivia (n = 6), Chile (n = 5), Uruguay (n = 1), Venezuela (n = 2) and Mexico (n = 1). Thirty studies were performed in non-endemic countries: Spain (n = 14, 9%), US (n = 13, 9%), Italy (n = 3) and the UK (n = 1).

Table 1 outlines the characteristics of included articles that presented any prevalence data for HIV/*T. cruzi* co-infection, either *T. cruzi* infection amongst subjects with HIV or HIV infection amongst individuals with *T. cruzi* infection or both.

Table 2 outlines the characteristics of included articles that presented aggregate data of selected populations only (i.e., did not meet the criteria of a prevalence study and/or presented no individual-level patient data).

Table 3 outlines the characteristics of included articles that had any individual-level data which could be extracted (and therefore include in our individual patient data analysis).

### Risk of bias in studies

The majority of articles were case reports or case series (94 articles, 62%), which are particularly susceptible to selection bias and publication bias. Additionally, the analysis of individual patient data may have unknowingly counted patients twice where authors have published multiple series, further contributing to risk of bias.

All 152 papers were critically appraised using the design-appropriate Joanna Briggs Institute (JBI) critical appraisal tool. Seven articles (5%) were assessed as high quality (a score above 90%), 83 articles (55%) medium quality (score of 75–90%), and the remaining 62 articles (41%) as low quality (score <75%). The majority of the cases reports and case series were of low quality. The mean JBI score for all included articles was of low quality, at 67%.

### Results of individual studies

#### Prevalence of co-infection.

Table 1 outlines study characteristics of four articles that presented prevalence of co-infection (and met the aforementioned criteria for prevalence studies). First-time blood donors in Mato Gross do Sul State of Brazil had a reported prevalence of *T. cruzi* HIV co-infection of 0.2% (Aguiar 1999) [14].

In the three studies of representative (outpatient) populations with HIV tested for *T. cruzi* infection, the prevalence of co-infection ranged from 1.3% in a study of 716 subjects in Sao Paulo, Brazil (Almeida 2010) [15] to 5% in a study of 200 subjects in Rio Grande do Sul State (Stauffert 2017) [16].

#### Proportion of selected study populations with co-infection.

In the four studies reporting testing of selected populations not previously known to have either *T. cruzi* or HIV, the proportion found to have co-infection ranged from 0.1% in emergency department attendees in Argentina (Auger 2002) [17] to 1% in migrants from Chagas-endemic countries in Spain (Bocanegra 2014) [18].

Twenty-three studies (that did not meet the criteria of a prevalence study) reported *T. cruzi* serological testing amongst people living with HIV (PLHIV). Excluding the nine studies where patients were explicitly selected based upon specific clinical presentations or risk groups (e.g., PLHIV with diarrhoea or neurological disease), the proportion testing positive for *T. cruzi* ranged from 0.2% in an outpatient setting in Brazil (Dias 2018) [19] to 28.6% in an inpatient setting in Bolivia (Reimer-McAtee 2021) [20].

Three studies, all from Brazil, reported testing for HIV in patients with known *T. cruzi* infection in hospital settings, amongst whom the proportion of patients with co-infection ranged from 26.4% (Sartori 2002) [21] to 47.9% (Perez-Ramirez 1999) [22].

Martins Melo et al. reported that both Chagas disease and HIV were mentioned on 196 death certificates in Brazil (out of 22,663,092) between 2000 and 2019 [23].

Twelve studies reported on testing of migrants from Chagas-endemic countries living in non-endemic settings. In the eight studies reporting testing for *T. cruzi* infection in PLHIV the proportion ranged from 1.1% in primary care in the US (Evans 2018) [24] to 10.5% in Spain (Rodriguez-Guardado 2009) [25].

#### Clinical features of co-infection.

One cross-sectional retrospective multicentre study of co-infection (Shikanai-Yasuda, 2021) [69] reported on 241 patients with co-infection (87% from Brazil), of whom 60 were cases of reactivation. In these 60 patients with reactivation, 35 (58%) presented with meningoencephalitis, 10 (17%) with myocarditis and eight (13%) with both. 31 (52%) had CD4 counts below 200 cells/mm^3^ at presentation. Mortality overall was 67%, higher in those with meningoencephalitis (77%), who on average had lower CD4 counts compared to myocarditis (in whom mortality was 50%). Although this article did not report individual-level patient data (and is therefore not included in our analysis), individual level data is available in an online repository and most of the individuals with reactivation referred to in this article have been published in a separate case series so are captured in our individual patient analysis.

This study did not report individual-level patient data so is not included in our analysis below, however most of the individuals with reactivation referred to in this article have been published in separate case series so are captured in our individual patient analysis.

110 studies reported individual-level patient data on clinical features or outcomes for a total of 352 patients with HIV and *T. cruzi* co-infection (once duplicate patients reported across multiple articles were excluded). Study characteristics are summarised in Table 3. Demographics of patients with HIV/*T. cruzi* co-infection are summarised in Table 4.

**Table 4:** Characteristics of 352 patients reported with co-infection (from 110 studies reporting individual-level patient data).

Age, median (IQR)	36 (30–44)
**Sex**	
Female	83 (35%)
Male	155 (65%)
Not reported	114
**Country of presentation**	
Brazil	177 (50%)
Argentina	88 (25%)
Bolivia	39 (11%)
Spain	21 (6%)
US	13 (4%)
Chile	4 (1%)
Colombia	3 (0.9%)
Italy	2 (0.6%)
Venezuela	2 (0.6%)
Mexico	1 (0.3%)
United Kingdom	1 (0.3%)
Uruguay	1 (0.3%)
Not reported	0
**Country of birth**	
Brazil	140 (40%)
Bolivia	55 (16%)
Argentina	49 (14%)
Honduras	5 (1%)
Chile	4 (1%)
El Salvador	4 (1%)
Mexico	4 (1%)
Colombia	3 (1%)
Paraguay	2 (0.6%)
Venezuela	2 (0.6%)
Ecuador	1 (0.3%)
Spain	1 (0.3%)
Uruguay	1 (0.3%)
Not reported	81 (23%)

Denominator for given percentages is total data available (excluding missing data).

#### Definition of co-infection and reactivation.

**Fig 3:**
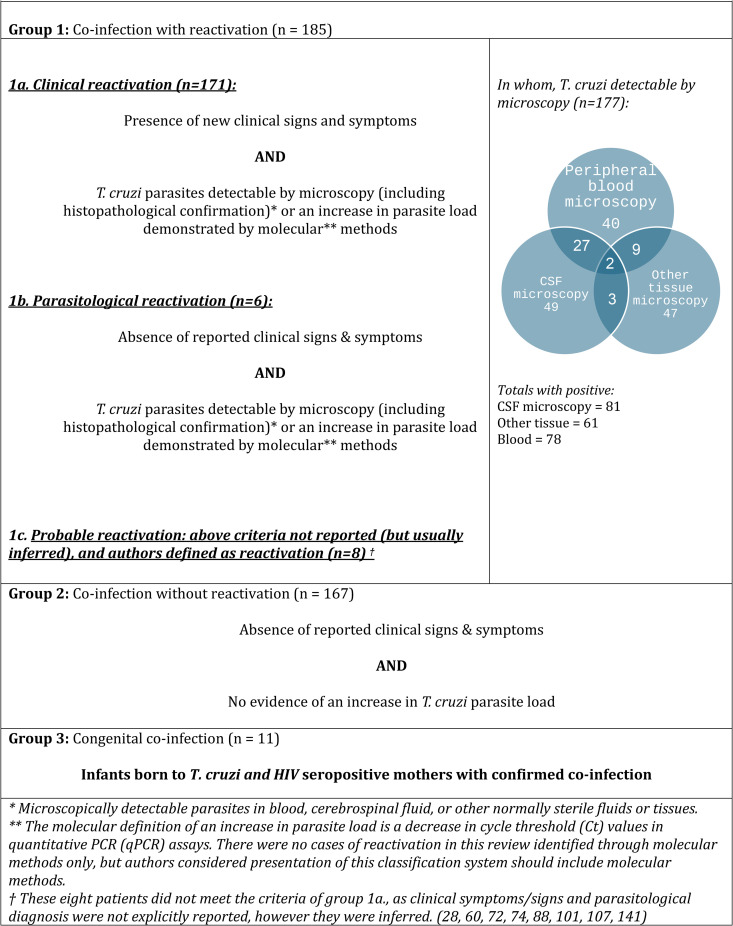
Clinical and parasitological classification of 352 patients with HIV and *T. cruzi* co-infection (and individual level data).

All 352 patients had laboratory-confirmed *T. cruzi* and HIV co-infection. There was a lack of an internationally standardised definition of *T. cruzi* reactivation in PLHIV and significant heterogeneity in the reporting of co-infection, with different clinical and laboratory parameters adopted historically and geographically. Therefore, for the purposes of this systematic review, individual cases were assigned one of three clinical phenotypes (co-infection with reactivation, co-infection without reactivation, and congenital co-infection. Fig 3).

### Individual patient analysis of *T. cruzi* reactivation

From here onwards, only the data from the 185 individuals who met our aforementioned ‘Co-infection with *T. cruzi* reactivation’ criteria (Group 1 of Fig 3) are reported. The 185 subjects determined as having reactivation had a median age of 36 (IQR 29–42). 95(51%) were male, 37(20%) female, and sex was not reported in 53 (29%) cases. 170 (92%) cases presented in endemic settings (39% of whom in Brazil), and 15 (8%) in non-endemic settings.

#### HIV and *Trypanosoma cruzi* status.

125 (68%) cases were known to have HIV prior to presentation, HIV was unknown at the point of presentation in 53 (29%) patients and was not recorded in 7 (4%) cases. 15 (8%) cases of reactivation occurred in patients on antiretroviral therapy (ART) (none of whom were HIV virally suppressed in the five cases where viral load was reported), 82 (44%) in ART-naïve cases and this data was missing in 88 (48%) cases. HIV viral load was reported in 31 cases of reactivation (median 143,000, IQR 26,168 –375,000, range 4,700 – 1,800,000 copies/mL), none of whom were virally suppressed. 45 (24%) cases of reactivation were known to have *T. cruzi* infection prior to presentation, this was unknown in 102 (55%) patients and not recorded in 38 (21%) cases. No cases of reactivation were noted to have previously received trypanocidal treatment with benznidazole or nifurtimox, but one case had received ketoconazole (published in 1999) [139].

#### Clinical presentation of reactivation.

Table 5 summarises the HIV and *T. cruzi* investigation findings of all cases of reactivation and Table 6 the presenting features.

39 (32%) individuals with reactivation were reported as acutely unwell (clinical shock – as defined by authors or systolic blood pressure <90 mmHg, altered consciousness or requiring critical care).

**Table 5:** HIV and *T. cruzi* investigation findings of 185 patients with presumed reactivation.

Investigations, units	Number of patients
(n result available/total)	
**CD4 count, cells/mm**^**3**^ **(96/186)**	
Mean (SD)	93 (112)
Median (IQR)	51 (19-110)
0-99	65 (68%)*
100-199	18 (19%)*
200-499	12 (13%)*
>=500	1 (1%)*
**HIV viral load, copies/mL (31/186)**	
Mean (SD)	289,293 (405,833)
Median (IQR)	143,000 (26,168-375,000)
***T. cruzi* parasite detectable by:**	**No. patients (%)**
Blood microscopy	78 (42%)^†^
Blood culture	20 (11%)^†^
Xenodiagnosis^***‡***^	24 (13%)^†^
Blood PCR	28(15%)^†^
CSF microscopy	81 (44%)^†^
CSF PCR	9 (5%)^†^
CSF culture	1 (0.5%)^†^
CSF serology	15 (8%)^†^
Biopsy	60 (32%)^†^
*Brain*	51 (28%)^†^
*Skin*	3 (2%)^†^
*Other*	4 (2%)^†^

*Denominator for given percentages is total data available for this variable (excluding missing data).

†Denominator for given percentages is total number of patients (185). ‡24 positive xenodiagnoses come from 15 papers, all in Brazil or Chile. PCR = polymerase chain reaction. CSF = cerebrospinal fluid.

**Table 6:** Clinical presentation of 185 patients with *T. cruzi* reactivation.

Presentation	Number of patients (%)
**Fever**	64 (35%)
**Weight loss**	29 (16%)
**Neurological**	
Focal deficit	71 (38%)
Weakness	65 (35%)
Headache	56 (30%)
Seizures	41 (22%)
Cognitive deficit	24 (13%)
Loss of consciousness	19 (10%)
Neck stiffness	17 (9%)
Speech disturbance	10 (5%)
Visual disturbance	10 (5%)
Tremor	2 (1%)
Any neurological presentation	157 (85%)
**Gastroenterological**	
Diarrhoea	10 (5%)
Vomiting	13 (7%)
Dysphagia	4 (2%)
Any gastroenterological presentation	27 (15%)
**Cardiorespiratory**	
Shortness of breath	10 (5%)
Cough	8 (4%)
Palpitations	1 (0.5%)
Chest pain	1 (0.5%)
Any cardiorespiratory presentation	18 (10%)
**Skin lesions**	5 (3%)

Denominator for percentages is total number of patients (185).

### Central nervous system reactivation

Meningoencephalitis and/or central nervous system (CNS) space-occupying lesions accounted for 85% of the cases of reactivation, with presentation with focal neurological deficit, headache or seizures most commonly reported, in 38%, 30% and 22% respectively.

Median CD4 counts in reactivation with CNS involvement were lower than those without CNS involvement (46 versus 102 cells/mm^3^, Wilcoxon rank-sum z statistic 2.12, p < 0.05).

92% of CNS reactivation cases had a CD4 count under 200 cells/mm^3^; 75% were under 100 cells/mm^3^.

This is a visual representation of the distribution of the data. The box represents the middle 50% of the data, from the first quartile to third quartile. The line inside the box represents the media and the x the mean. The whiskers extending from the box show the range of the data, extending to the smallest and largest values within 1.5 times the interquartile range from the box. Data points that fall outside the whiskers are considered outliers and are shown as individual points.

#### Cerebrospinal fluid examination.

**Fig 4:**
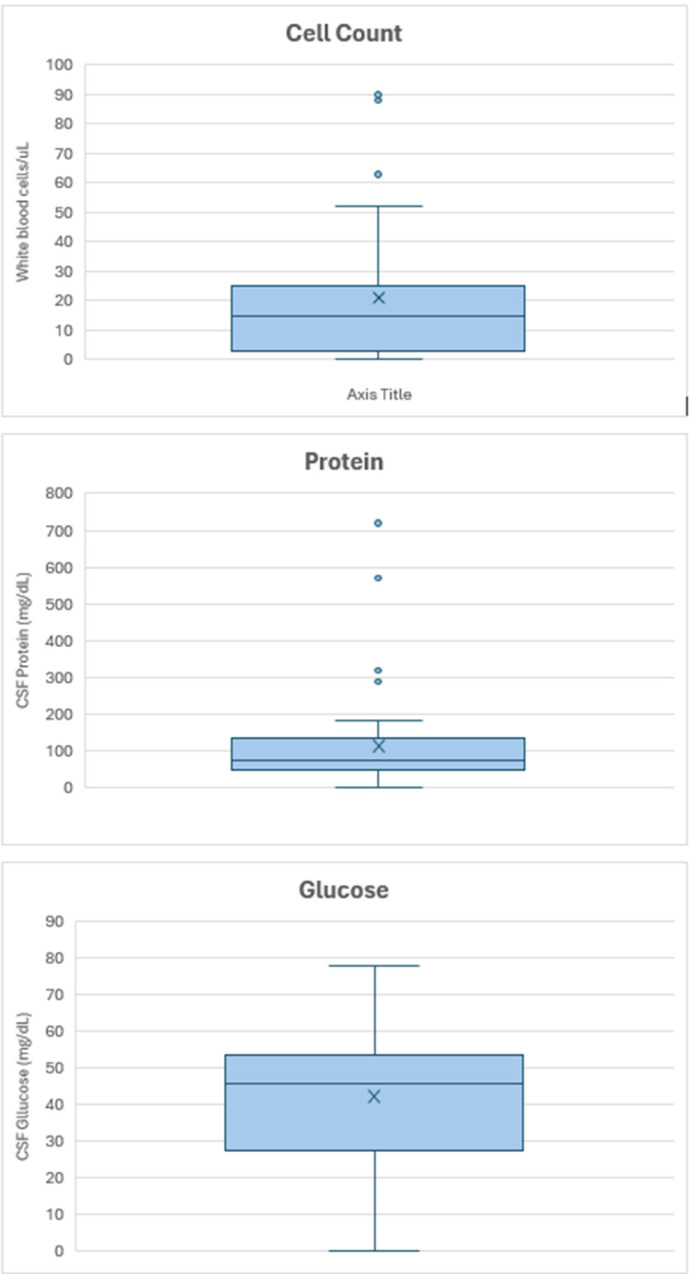
Box and whisker plots of cerebrospinal fluid examination findings in patients with central nervous system *Trypanosoma cruzi* reactivation (a. White cell count, b. Protein and c. Glucose).

104 (56%) patients with reactivation had a lumbar puncture (LP), of whom 98 had neurological symptoms and/or abnormal neuroimaging findings reported suggestive of CNS pathology. The cerebrospinal fluid (CSF) was reported as normal in five cases (one of whom had reported symptoms or neuroimaging suggestive of CNS reactivation) and abnormal in 97 cases (95 of whom had neurological symptoms and/or abnormal neuroimaging findings reported suggestive of CNS reactivation and two of whom did not). *T. cruzi* was detectable by microscopy in the CSF in 80 cases (83% of presumed CNS reactivation cases with CSF findings reported, Table 4). In three cases, *T. cruzi* was not detectable by CSF microscopy, but CSF PCR was positive. Fig 4 displays the distribution of CSF white cell counts, protein and glucose, for patients with CNS reactivation. Data on CSF findings pertaining to other OIs was not systematically extracted.

Of 41 reported CSF cell counts, the cell count was greater than five white blood cells/µL in 61% of cases (greater than 10 in 54%, greater than 20 in 32%, greater than 50 in 15% cases) and, when reported, was predominantly lymphocytic.

According to a normal protein CSF reference range of 15–60 mg/dL, of 44 reported protein values: 26 (59%) were raised (48% greater than 80, 34% greater than 100, 10% greater than 200).

According to a normal glucose CSF reference range of 50–80 mg/dL, of 37 reported values, 23 (62%) reported low glucose and 14 (38%) reported normal glucose.

#### Neuroimaging.

122 patients had neuroimaging: 104 patients (56%) had a CT brain, 47 (25%) an MRI and 29 (16%) had both. In six symptomatic patients, neuroimaging was reported as normal (all six patients had an LP, five of which were reported – all abnormal, including three which had *T. cruzi* detectable by microscopy in the CSF and one with PCR positive CSF). 53 (43% of those with neuroimaging) patients had a single lesion, and 61 (50%) patients had multiple (2 or more) lesions. 47 (39%) lesions were reported as ring-enhancing (and it should be noted that use of contrast was often not reported), eight (7%) reported as located in white matter, three (2%) in grey matter (and location not stated in the majority of reports). Cerebral oedema was present in 53 (43%), with a mass effect in 37 (30%).

### Cardiac reactivation

Myocarditis was reported in 18 cases (10% of all reactivation reports) and was a common finding on autopsy (12 out of 35 cases, 34%). The median CD4 count of the 11 cases for whom CD4 count was reported was higher (median 157, [IQR 104–264]) than for those with CNS reactivation (Wilcoxon rank-sum z = -3.13 p < 0.01).

#### Cardiorespiratory investigations.

In the absence of prior data on cardiac function, attribution of reported cardiac abnormalities to *T. cruzi* reactivation cannot be substantiated, nevertheless the reported findings (for all patients with presumed reactivation) were as follows: ECG was reported as abnormal in 28 (15%) cases (supraventricular tachycardia = 3, ventricular tachycardia = 1, right bundle branch block = 7, left bundle branch block = 1, atrioventricular block = 8, ventricular ectopics = 1, ST changes = 1, prolonged QT = 1). Chest x-ray was reported as abnormal in 17 (9%) cases (cardiomegaly = 7, pneumonia = 5, infiltrates = 5, effusion = 4). Echocardiography was reported as abnormal in 16 (9%) cases (systolic dysfunction = 9, thrombus = 1, valve disease = 2, pericardial effusion = 5, dilatation = 4).

### Other clinical presentations

Other clinical presentations of *T. cruzi* reactivation in PLHIV were less frequently reported, and included, but were not limited to, erythema nodosum in two cases [60,75,152], and pleuritis [140], peritonitis [124], cervicitis [159] and panniculitis [75] in one case each.

### Opportunistic infections

Concurrent additional opportunistic infections were commonly reported (n = 67, 36%). Herpes simplex virus in four cases (2%), cryptococcosis in six cases (3%), tuberculosis in 11 cases (6%), cytomegalovirus in 13 cases (7%), candidiasis in 14 cases (8%), pneumocystis pneumonia in 13 cases (7%), and toxoplasmosis in 34 cases (18%). In the cases of concurrent cryptococcosis, cytomegalovirus and toxoplasmosis, CNS involvement (of these other OIs) was usually inferred but rarely well described.

### Management

Anti-retroviral treatment (ART) was poorly reported. ART was initiated in 43 (23%) patients at some point (43/185, 111 had missing data). Delaying initiation of ART was explicitly noted in 15 (8%) cases. 124 patients (67%) were treated for reactivation disease with trypanocidal drugs (107 with benznidazole and 17 with nifurtimox). Adverse effects from trypanocidal drugs were reported in 22 patients (18% of those treated), with 11 of those 22 patients having to stop trypanocidal treatment because of this. The mean trypanocidal treatment duration was 60 days (standard deviation 104, range 1 – 730). 55 (30%) patients were treated for toxoplasmosis. Steroid therapy was given in 34 (18%) cases. Data on ART choice and timing, as well as secondary (trypanocidal) chemoprophylaxis were lacking. Immune reconstitution inflammatory syndrome (IRIS) was reported (as probable, rather than confirmed) in two cases [74,144], both of whom died.

### Outcomes

Overall mortality was 67% (124 patients died out of 185 with reactivation). Seven (4%) survived with morbidity (of which five had neurological sequelae, one had congestive cardiac failure and one not described). Thirty-four patients (18%) fully recovered whilst outcome data was missing for 20 (11%) patients.

Overall mortality in patients with CNS reactivation (79%) was higher than those without (54%) CNS reactivation (*χ*^2^ = 7.5, p < 0.01)

Amongst those with data available on both ART at presentation and clinical outcome, there were 7 deaths in 15 people reported to be on ART at presentation (47% mortality), compared to 54 deaths in 77 people known to not be taking ART at presentation (70% mortality).

Time to death was reported in 71 cases, amongst whom it occurred at a median of 21 days after presentation (IQR 8–60 days, range 0–1,950). Median time to death in cases of CNS reactivation was shorter (21 days, IQR 8–57) than for those without CNS reactivation (41 days, IQR 14–75), but this was not statistically significant.

In univariate analyses, age, sex and CD4 count were not statistically significant predictors of death, but neurological presentation was (*χ*^2^ = 7.5, p < 0.01).

### Autopsies

38 autopsies were reported in people known to have HIV/*T. cruzi* co-infection (35 in cases of reactivation). The macro- and microscopic patterns of necrosis, haemorrhage and inflammation of cerebral lesions, and direct observation of *T. cruzi* parasites, were described in 15 articles [32,60,78,89,92,93,100,103,139,142,144,148,155,157,162]. Carditis (mostly myocarditis with amastigotes in muscle cells with infiltrates) was reported in 12 articles [84,89,92,100,107,108,118,125,139,152,154,155]. Nests of *T. cruzi* amastigotes with inflammation, necrosis and haemorrhage in the adrenal glands were reported in one article [79]. Myositis of the extra-ocular muscles was reported in one article [142].

### Maternal and congenital co-infection

Eight articles reported maternal and/or congenital co-infection [51,62,90,105,111,147,150,154]. Scapellato [62] assessed *T. cruzi* vertical transmission rates in 94 *T. cruzi* seropositive mothers (three of whom were co-infected with HIV). All three neonates born to co-infected mothers had congenitally acquired *T. cruzi* (versus 10 out of 91, 10.9%, of those born to HIV negative mothers). Similarly Nisida [154] reported *T. cruzi* vertical transmission rates in 58 *T. cruzi* seropositive mothers: congenital *T. cruzi* transmission occurred in both infants born to the two HIV co-infected mothers compared to two out of 56 (4%) born to HIV negative mothers.

The outcomes of eight cases of co-infection in pregnant women (of which three were reactivation, all survived) were reported [26,60,150,154]. This included four cases published by Sartori et. al in which three resulted in congenital transmission. All three neonates were symptomatic with CNS disease (and two out of three neonates died) [60]. One case report of *T. cruzi* CNS reactivation in a pregnant woman with co-infection reported the use of benznidazole at 32 weeks in successfully treating the woman; congenital infection did not occur [150].

Eleven congenital cases of co-infection were reported in six articles [51,90,105,111,147,154], of whom nine were symptomatic, with meningoencephalitis (n = 5), anaemia (n = 3) and/or hepatosplenomegaly (n = 3), and six died (of which three had neurological involvement and two with sepsis).

## Discussion

This systematic review provides the most comprehensive collation and overview of published evidence on the epidemiology and clinical manifestations of HIV and *Trypanosoma cruzi* co-infection and reactivation. Although this review includes a large body of evidence (152 articles published over 34 years, reporting on 352 co-infected patients with individual data from 12 countries), the granularity of data in the majority of published articles was low and missing data on key variables was common. As outlined by Clark et al. in a 2021 narrative review on Chagas disease in people with HIV [10], we found significant heterogeneity in the way *T. cruzi* reactivation is reported across articles. The lack of any internationally recognised and standardised definition of reactivation hindered the interpretation of our results. To aid future research and guideline development, we recommend clearer reporting of clinical and diagnostic (parasitological and molecular) criteria for *T. cruzi* reactivation. Expert guidelines are clear that microscopic detection of *T. cruzi* is diagnostic of reactivation, [164,165] however there is no clear consensus on a threshold for parasite load as detected by molecular methods (and development of such consensus is hindered by the lack of standardised PCR procedures for *T. cruzi*).

Despite advances in access to, and efficacy of, antiretroviral therapy [166,167] reported in the literature and downward trending incidence of *T. cruzi* infection, the proportion of co-infection reported in hospitalised populations remained high in the more recent publications, for example 24% and 38% of hospitalised patients with known HIV screened for *T. cruzi* in two different hospitals in Bolivia, published in 2015 and 2021 respectively [20,39]. As no cases of reactivation identified in this review occurred in individuals on ART who were virally suppressed, reactivation cases in modern times are more likely due to access to ART issues rather than their lack of efficacy in preventing reactivation. In one study from Argentina published in 2016, other HIV risk groups with high *T. cruzi* seroprevalence were men who have sex with men, STI clinic attendees and intravenous drug users, with an overall co-infection prevalence of 3% [40]. Our findings support the recommendation for universal co-infection screening for those known to have either HIV or *T. cruzi* in endemic settings, ideally at the first point of contact with healthcare providers.

The proportion of co-infection reported in studies performed in non-endemic settings varied more widely, from 1-2% when screening Latin American migrants with HIV for *T. cruzi* infection in Italy, the US and the UK [24,45,46], to 3–11% in Spain [25,26,44]. Some study authors attributed lower than expected proportions to poor uptake of testing, especially amongst groups born in higher-endemicity countries. Low awareness of Chagas disease amongst at-risk migrants, as well as healthcare professionals in non-endemic countries, has been reported in the literature as a potential barrier to screening uptake [168–171]. We recommend educational interventions, which have successfully been implemented, to improve uptake of screening in these settings [172], whilst acknowledging that the demographic profile of migrant communities from Latin America at highest risk of *T. cruzi* infection may not overlap substantially with that of migrant populations at highest risk of HIV infection.

This review identified very little evidence on maternal and congenital HIV-*T. cruzi* co-infection, and this is recommended as a priority area for future research. The eight published articles on this topic suggest high rates of congenital *T. cruzi* infection amongst neonates born to mothers with co-infection (compared to mothers with *T. cruzi* alone). The articles also suggest very high neonatal mortality associated with co-infection.

Risk of *T. cruzi* reactivation in immunosuppression was assessed in another recent systematic review by Antequera et al. [173], which included three prospective studies of HIV/*T. cruzi* co-infection.[20,40,60] The incidence of reactivation ranged from 11% to 26%, with a pooled cumulative incidence of reactivation of 17% (95% CI 8–29%) in PLHIV, which was lower than other contexts of acquired immunosuppression such as transplant recipients. In our review, we felt the level of heterogeneity between both study contexts and populations, as well as the different interpretations of what constitutes reactivation, plus the limited follow-up time in any prospective studies (which were lacking altogether) impeded a potential meta-analysis of risk of reactivation. More research is needed to understand the true risk of reactivation (including the effect of trypanocidal drugs), with a clearly defined study population and definition of reactivation, prospective outcome measurement, and sufficient sample size and follow-up duration.

This systematic review’s individual-patient analysis focused on 186 cases of co-infection with *T. cruzi* reactivation. The majority of these patients were born in and presented to healthcare systems in Brazil, with a small representation of patients additionally from Argentina and Bolivia, and a small number of migrants from Bolivia, El Salvador or Honduras presenting in the US or Spain.

Reactivation was mostly reported in patients with a CD4 count below 200 cells/mm^3^ (87% of patients), 68% were lower than 100. In our review, we found that CNS reactivation accounts for 85% of the total cases of reactivation and is associated with lower CD4 counts (only one case occurred in those with CD4 counts over 500 cells/mm^3^). Our findings on the clinical features of *T. cruzi* CNS reactivation are similar to those of a recently published review on this topic which found most (but not all) cases of CNS reactivation to occur in individuals with CD4 counts below 200 cells/mm^3^, a similar proportion of patients presenting with seizures and motor deficits, and a similar mortality overall (74%) [174]. Our systematic review is more comprehensive, in that a more exhaustive search strategy was utilised, thus identifying more cases of reactivation. HIV viral load was underreported (only 31 cases reported this), nonetheless all 31 cases showed detectable viral loads. As has been previously reported [10], this review found CNS lesions from *T. cruzi* reactivation to be mainly (but not exclusively) ring-enhancing, and cerebral oedema was commonly reported. Expert groups with significant clinical experience in this area have reported that *T. cruzi* lesions tend to occur in white or subcortical matter, by contrast with Toxoplasma which may preferentially affect the thalamus and basal ganglia [175], however this was not clearly substantiated from our systematic review. As there are reports (n = 6) of normal neuroimaging in *T. cruzi* CNS reactivation, CSF examination (ideally including *T. cruzi* PCR as per expert guidance [165,175]) is recommended in cases of sufficient clinical suspicion, even if imaging is reported as unremarkable.

Neuroimaging can be normal in *T. cruzi* CNS reactivation. If *T. cruzi* reactivation is suspected, direct microscopic examination of the CSF is recommended. Where available the use of PCR in CSF to enhance sensitivity has been recommended in expert guidelines [165,175]. Further research to assess the extent to which PCR enhances sensitivity over microscopy is recommended, given that access to PCR remains limited in low-resource settings. Alongside the other main differential diagnoses for PLHIV presenting with neurological features (toxoplasmosis, primary CNS lymphoma, progressive multifocal leukoencephalopathy, tuberculosis and cryptococcosis for example), we recommend that *T. cruzi* should be considered in anyone who has spent time in any of the 21 Chagas-endemic countries of South America, Central America or Mexico (and people born to mother from endemic countries).

We found a smaller proportion of cardiac reactivation (namely myocarditis) –10% of published cases of reactivation, than expert review articles have suggested (30–40%) [175]. As has previously been postulated, the burden of Chagasic cardiac reactivation is difficult to quantify as it may go unrecognised if not severe, that is, it could be attributed to chronic determinate disease. Additionally non-fatal cases are less likely to be published.

Robust data on management and outcomes of *T. cruzi* reactivation was lacking, although the lack of published reports of immune reconstitution inflammatory syndrome (IRIS) suggest this is not a common feature. However, it is possible that IRIS is not well described in these cases due to patients dying before IRIS manifested. Expert guidelines suggest waiting until at least three weeks of treatment with benznidazole (or nifurtimox) has been received prior to starting ART [175]. Although the evidence-base is lacking, expert guidelines also recommend secondary chemoprophylaxis for patients who have experienced reactivation due to *T. cruzi*/HIV co-infection when CD4 level is below 200 cells/mm^3^ (but do not recommend primary prophylaxis) [175].

The main limitation of the body of evidence presented in this systematic review stems from the biases (namely selection bias and publication bias) to which case series are susceptible. Severe and fatal cases are more likely to be published, and the proportion of reactivation presenting with neurological symptoms may be overrepresented as they are more likely to be attributed to *T. cruzi* reactivation. Although we were able to estimate the number of cases of co-infection and reactivation (with individual level data) ever published in the literature, it is a limitation of this paper that there was no feasible way to calculate the total cases from aggregate data. One retrospective multicentre study of co-infection was identified, which reported on 241 patients with co-infection, mainly from Brazil, and it is a further limitation of our review that we could not extract individual level patient data from this study to include in our analysis (however it is thought most of the cases of reactivation were published elsewhere so have been captured in our search).

## Conclusion

Most evidence on HIV/*T. cruzi* co-infection and reactivation comes from case series, which are susceptible to selection and publication biases and therefore mainly of low quality. Evidence on maternal and congenital co-infection is particularly lacking. From the published literature, *T. cruzi* reactivation mainly affects those with untreated HIV and lower CD4 counts. CNS reactivation is the most common clinical picture, typically causing meningoencephalitis and cerebral lesions, and confers high mortality. Prompt recognition of reactivation and immediate initiation of trypanocidal therapy (with benznidazole or nifurtimox) is recommended. Screening for co-infection should be implemented in endemic settings (for *T. cruzi* in those with HIV and vice versa) and for migrants from at-risk areas (and those born to mothers from endemic countries) in non-endemic settings. Educational initiatives are needed to increase healthcare professionals’ awareness of Chagas disease, in particular of the risk of reactivation in HIV and other forms of immunosuppression.

## Supporting information

S1 Checklist PRISMA Checklist. Legend: This article was reported according to PRISMA guidelines, as outlined in this checklist (available at: https://www.prisma-statement.org/prisma-2020-checklist).
(DOCX)
